# The Relationship Between Lymphocyte Subtypes with Clinicopathological Features and Prognosis of Prostate Cancer in Patients Undergoing Radical Prostatectomy

**DOI:** 10.5152/tud.2023.22220

**Published:** 2023-07-01

**Authors:** Furkan Sendogan, Turgay Turan, Hayriye Erman, Yavuz Onur Danacioglu, Ferruh Kemal Isman, Ramazan Gokhan Atis, M. Selcuk Silay, Turhan Caskurlu, Asif Yildirim

**Affiliations:** 1 Department of Urology, Şişli Memorial Hospital, Istanbul, Turkey; 2 Urology Clinic, Turgutlu State Hospital, Manisa, Turkey; 3 Department of Medical Biochemistry, Istanbul Medeniyet University, Istanbul, Turkey; 4 Urology Clinic, Health Sciences University Bakırköy Dr. Sadi Konuk Training and Research Hospital, Istanbul, Turkey; 5 Department of Urology, Istanbul Medeniyet University, Istanbul, Turkey; 6 Department of Urology, Ataşehir Memorial Hospital, Istanbul, Turkey

**Keywords:** Lymphocyte, prognosis, prostate cancer, radical prostatectomy

## Abstract

**Objective:**

The immune system has an essential role in the development of cancer by showing both anti-tumor and pro-tumor activities. Understanding the immune function of patients with malignancy is of clinical importance for the evaluation, treatment, and prognosis of the disease. We aimed to evaluate lymphocyte subtypes in peripheral blood samples of prostate cancer patients and their relationship with clinicopathological features and prognosis.

**Methods:**

One hundred thirty-seven patients who underwent open radical prostatectomy were included in our study. The percentages of CD3+T lymphocyte, CD19+ B lymphocyte, CD16/56 natural killer cells, CD4+ helper T lymphocyte, CD8+ cytotoxic T lymphocyte, and CD45 total lymphocyte were evaluated for each patient using the blood sample taken into a hemogram tube before surgery.

**Results:**

The pathological stage was T2 for 64 of the cases and T3 for 73. The mean follow-up period of the patients was 12.81 ± 6.20 months. The CD3+/CD4+ counts of the patients with pathological stage T2 were found to be statistically significantly higher than stage T3. There was a statistically significant negative correlation between the prostate-specific antigen levels and CD3+/CD4+ percentages of the patients. There was no statistical significance between the percentages of lymphocyte subtypes and the presence of surgical margin, biochemical recurrence, adjuvant therapy, and cancer upgrade.

**Conclusion:**

We consider that the increase in the pathological stage and prostate-specific antigen value and the decrease in the number of CD4+ T lymphocyte subtypes may be prognostic markers in prostate cancer patients.

**Main Points**

- Current research suggests a relationship between preoperative CD4 + T lymphocyte count, prostate-specific antigen value, and pathological stage.
- Lymphocyte subsets, especially CD4 T lymphocyte, might be potential prognostic biomarkers of prostate cancer.
- Many nomograms used to predict the prognosis of prostate cancer have been described in the current literature.
- In future studies, CD4 T lymphocyte ratio in peripheral blood can be added as a parameter of a nomogram that can predict prostate cancer prognosis.

## Introduction

Prostate cancer (PCa) is the second most commonly diagnosed cancer in men, and it is estimated that approximately 248 000 people in the United States will be diagnosed with prostate cancer and there will be 34 000 cancer-related deaths in the United States of America by the end of 2021.^1^ There are many studies in the literature providing new information and the underlying etiology about PCa.^2,3^ Although family history and ethnicity are associated with an increased incidence of PCa, only about 9% of patients have truly hereditary disease.^4^ In addition to the genetic background, many exogenous and environmental risk factors have been investigated in terms of their roles in prostate cancer development and progression, and it has been discussed in the current literature that some of these factors may be associated with PCa.^5^ In a meta-analysis, Dennis et al^2^ stated that prostatitis or sexually transmitted infection might be associated with PCa, while in another study, De Marzo et al^6^ reported that patients with PCa had higher plasma concentrations of proinflammatory cytokines and acute phase reactants than healthy individuals. This previous research led to the investigation of the link between an inflammatory microenvironment and tumor development based on clinical, molecular, histopathological, and epidemiological evidence. Although the role of the immune system in the development of PCa cannot be clearly defined, lymphocyte subtypes are considered to play an important role in the local or systemic development and progression of PCa.^6-8^

In our study, we investigated the relationship between lymphocyte subtypes, such as CD3, CD16/56, CD19, CD4, and CD8 in the peripheral blood before treatment and histopathology results and prognosis in patients diagnosed with PCa.

## Material and Methods

### Patient Selection

The study was approved by the Ethics Committee of Istanbul Medeniyet University (Approval date: April 18, 2017, decision number: 2017/0152). Between October 10, 2016, and December 10, 2018, patients who were diagnosed with 184 prostate cancer patients were evaluated. Twelve patients were diagnosed with prostate cancer but decided on active surveillance or watchful-waiting, 25 patients who underwent radiotherapy for definitive therapy, 8 patients with metastasis at the time of diagnosis, 1 patient who has an autoimmune disease, and 1 patient with concurrent second primary cancer were excluded from the study. All of the patients gave study-specific informed consent. A total of the remaining 137 patients were included in the study. For each patient, a blood sample was taken into a hemogram tube before surgery.

### Surgical Procedure

The open radical prostatectomy technique was based on the principles proposed by Eastham and Scardino from the Memorial Sloan Kettering Cancer Center.^9^ Extended lymph node dissection was performed in patients with positive regional lymph nodes detected in imaging modalities or during the operation and in patients with an estimated probability of lymph node invasion over 5% according to the Partin nomogram.^10^

### Biochemical Analysis of Blood Samples

Lymphocyte subgroups (CD4, CD8, CD3, CD19, CD16, CD56, and CD45) were studied using the flow cytometry method in blood samples taken for analysis. The FACS CANTO II model of the Becton Dickinson flow cytometer was used for immunophenotyping. Lymphocyte subgroups were stained with fluorochrome dyes according to surface antigens before the procedure. CD4 surface antigen phycoerythrin cyanine 7, CD8 surface antigen allophycocyanin (APC), CD3 surface antigen fluorescein isothiocyanate, CD19 surface antigen peridinin-chlorophyll proteins, CD16-56 surface antigen phycoerythrin, and CD45 surface antigen APC-H7 were stained with fluorochrome. The stained lymphocyte surface antigens were passed through the flow cell meter. The cells were divided into groups according to the intensity of scattered fluorescence, and thus lymphocyte subgroup percentages were obtained.

Weight, height, body mass index (BMI), age, prostate-specific antigen (PSA) value, clinical T stage, D’Amico risk classification, transrectal ultrasound biopsy results, and retropubic radical prostatectomy (RRP) pathology results were recorded for all the patients included in the study. In addition, the presence of biochemical recurrence, time to recurrence, presence of clinical progression, presence of cancer upgrade, time to progression, type of adjuvant therapy applied (chemotherapy, radiotherapy, androgen deprivation therapy (ADT), or their combinations), and current survival status were evaluated. Aspartate aminotransferase (AST), alanine aminotransferase (ALT), hemoglobin, thrombocyte, neutrophil, lymphocyte, and leukocyte values were also recorded in routine blood tests taken during surgical preparations. The percentages of CD 16/56 NK cells, CD3+ T lymphocytes, CD19+ B lymphocytes, CD4+ helper T lymphocytes, CD8+ cytotoxic T lymphocytes, and CD45 total lymphocytes were evaluated for each patient using the blood sample taken into a hemogram tube 1 day before surgery. The patients were grouped according to the pathological stage, biochemical recurrence, cancer upgrade, adjuvant treatment, and surgical margin status, and the differences between lymphocyte subtypes were compared.

### Statistical Analysis

The Number Cruncher Statistical System (NCSS) 2007 (Kaysville, UT, USA) was used for statistical analysis. Descriptive statistical methods (standard deviation, mean, median, frequency, percentage, maximum, and minimum) were used when evaluating the study data. The conformity of quantitative data to normal distribution was tested with the Shapiro–Wilk test and graphical examinations. The independent-sample *t*-test was used for the comparison of normally distributed quantitative variables between 2 groups, and the Mann–Whitney *U* test was used for the comparison of 2 groups in terms of non-normally distributed quantitative variables. Pearson’s and Spearman’s correlation analyses were undertaken to evaluate the relationships between quantitative variables. Statistical significance was considered *P* < .05.

## Results

One hundred and thirty-seven patients who were admitted to our urology clinic and were diagnosed with PCa were included in the study. The demographic and clinicopathological features of the patients are described in Table 1. The pathological stage was T2 in 46.7% (n = 64) of the cases and T3 in 53.3% (n = 73). More specifically, the pathological stage was T3a in 35.8% (n = 49), and T3b in 17.5% (n = 24). According to the RRP pathology results, the tumor area of the patients ranged from 0.5% to 100%, with a mean value of 22.31 ± 22.24%. The surgical margin was positive in 21.2% (n = 29) of the cases and negative in 78.8% (n = 108) (Table 1).

No statistical significance was found between patient age and CD3+, CD 19+, CD3+/CD8+, and total lymphocyte ratio (*P* > .05). There was a positive correlation of 23.3% between patient age and CD 16/56+ measurements, which was found to be statistically significant (*r* = 0.233; *P* = .006; *P* < .01). In addition, a negative correlation of 17.2% was observed between patient age and CD3+/CD4+ measurements (as the former increased, the latter decreased), which was also statistically significant (*r* = −0.172; *P* = .044; *P* < .05).

Biochemical recurrence was observed in 26.3% (n = 36) of the cases. Time to recurrence ranged from 1 to 24 months, with a mean value of 9.90 ± 6.73 months. Clinical progression was observed in 2.2% (n = 3) of the cases. Time to progression ranged from 3 to 10 months with a mean value of 5.67 ± 3.79 months. Adjuvant treatment was applied to 25.5% (n = 35) of the patients, of whom 9 (6.5%) received ADT, 6 (4.3%) radiotherapy, 1 (0.7%) chemotherapy, and 19 (13.8%) ADT + radiotherapy. The median follow-up period of the cases was 12.81 ± 6.20 months.

Table 2 presents the percentages of lymphocyte subtypes in the peripheral blood of the patients.

There was no statistically significant correlation between BMI, lymphocyte subtype percentages, and total lymphocyte ratio of the patients participating in the study (*P* > .05) (Table 3).

No statistically significant correlation was found between Gleason score, lymphocyte subtype percentages, and total lymphocyte ratio of the patients participating in the study (*P* > .05). While no statistically significant correlation was observed between PSA levels and CD 3+, CD19+, CD3+/CD8+ measurements and total lymphocyte ratio (*P* > .05), there was a negative statistically significant correlation between PSA and CD3+/CD4+ values (*r* = −0.196; *P* = .022; *P* < .05) (Table 3).

The CD19+, CD3+, CD16/56+, and CD3+/CD8+ measurements and total lymphocyte ratio of the cases did not show a statistically significant difference according to pathological stage (*P* > .05). The CD3+/CD4+ values of the patients with pathological stage T2 were found to be statistically significantly higher than those with pathological stage T3 (*P* = .048; *P* < .05) (Table 4).

One hundred and one patients (73.7%) did not have biochemical recurrence while recurrence was observed in 36 cases. The mean recurrence-free survival was 18.18 ± 0.83 months. The last recurrence was seen at 12 months, at which the cumulative survival rate was calculated as 70.7% with a standard error value of 4.3%.

## Discussion

In this study, it was determined that there was a decrease in the level of helper T lymphocytes, PSA levels, and pathological stage in peripheral blood samples collected from patients with PCa. T lymphocytes have an important place in the formation of an immune response and provide the regulation of the cellular and cytokine-mediated interaction cascade. Previous studies revealing T lymphocyte functions have led researchers to further investigate this issue and to clarify the relationship between many types of cancer and the immune system, and this has led to various debates. Pardoll et al^11^ reported that CD4 T lymphocytes both stimulated and inhibited the immune system. Other studies have also defined the function of CD4 T lymphocytes as a “double-edged immunological sword,” since they play a key role in initiating and maintaining the anti-cancer immune response.^11-13^

The relationship between lymphocyte subtypes in peripheral blood and urological malignancies has also been evaluated. In one of the studies, Shaw et al^14^ reported that the presence of cancer and advanced stage caused a relative decrease in CD4 T lymphocyte activity compared to the healthy control group, and this effect was reversed in those receiving androgen suppression therapy. In the PCa group of the study, 37 patients were evaluated. It was stated that CD4 T lymphocyte activity was lower in patients with advanced PCa compared to the healthy control group, and this was statistically significant.^14^ In another study, in which 50 patients with PCa were evaluated in terms of lymphocyte subgroups, Oluboyo et al^15^ detected a significant decrease in CD8 and CD4 T lymphocytes in peripheral blood in the PCa group compared to the healthy control group. However, in the same study, no significant difference was found between the CD4, CD8, and CD4/CD8 percentages of patients according to stage and presence of chemotherapy.^15^ In our study, the percentages of lymphocyte subtype were examined in the peripheral blood sample of 137 patients with PCa, and the CD4+ T lymphocyte values were found to statistically significantly decrease with the increasing PSA value and pathological stage. The significant relationship between these parameters and CD4 T lymphocytes, which play a key role in the diagnosis, treatment, and follow-up of PCa, suggests that they can be used as important prognostic markers. Controlling the helper T lymphocyte level during the treatment decision phase can make a positive contribution to accurate staging. The detection of low helper T lymphocyte levels can additionally contribute to the decision of active surveillance, radiotherapy, or surgery. Recently, Mao et al^16^ evaluated peripheral blood lymphocyte subtypes and clinical outcomes of 135 prostate cancer patients and reported that an absolute CD4 T lymphocyte count of less than 255/μL is an unfavorable prognostic factor for prostate cancer survival. However, the remarkable part of the study is that only 35 patients underwent surgery and 72 patients were in the metastatic stage. In addition, 100 patients received treatments that affect the lymphocyte count such as endocrine therapy, chemotherapy, and radiotherapy. In our study, metastatic patients and patients who received additional treatment for prostate cancer were excluded from the study because it may affect the percentage of lymphocyte subtypes. In this respect, we think that the inclusion criteria of our study lead to more accurate results.^16^

Several studies have found that immune system cells infiltrate tumor cells at different stages of PCa. In a study evaluating the immune infiltration of PCa, it was reported that the diffuse lymphocyte infiltration of the tumor provided higher survival compared to lower infiltration.^17^ In addition, there are studies showing that high numbers of CD4 T lymphocyte invasions are associated with poor disease outcomes, such as postoperative biochemical recurrence and cancer-specific death.^17,18^ In a study by Rui et al,^19^ the T-helper-2 infiltration of immune cells was found to be associated with the recurrence of PCa. Similarly, Cequeira et al^20^ detected a decrease in CD4+ T lymphocyte infiltration in tissues after PCa cryoablation treatment. There is also research examining lymphocyte subtypes in peripheral blood samples after radiotherapy-brachytherapy performed for the treatment of PCa. It has been reported that lymphocyte subtypes may have prognostic significance in the clinical course of PCa after treatment.^21,22^

Recently, the tumor microenvironment has become one of the most discussed topics. The tumor microenvironment is composed of surrounding immune cells, fibroblasts, lymphocytes, bone marrow–derived inflammatory cells, extracellular matrix, blood vessels, and signaling molecules. It is considered that the interactions between non-malignant and malignant cells that constitute the tumor microenvironment play a central role in cancer development and progression.^23,24^ In tissue-based studies, changes in lymphocyte infiltration before and after treatment may be due to local effects or changes in the tumor microenvironment. In our study, we detected a decrease in the number of CD4 T cells in peripheral blood in patients with advanced PCa. This situation can be considered as a result of the systemic effect of the tumor due to the progression of cancer.

Prostate cancer and immunology-based studies, which are especially emphasized in the literature, have revealed the idea that immunotherapies can be used in the treatment of prostate cancer. In 2010, Smith et al^26^ in a phase 3 study conducted by 512 metastatic castration-resistant PCa patients; it has been stated that Sipuleucel T treatment, an autologous PCa vaccine, provides a survival advantage. Therefore, it is recommended by the current European guidelines with a strong degree of recommendation for the treatment of patients with castration resistance metastatic PCa.^25,26^ In a study by Fong et al,^27^ it was stated that there was an increase in the level of T cells in the peri-tumoral area in patients receiving Sipuleucel-T treatment. The authors noted that the majority of the T-cell population, especially CD4+ helper T cells, was the result of the effect of this treatment.^27^ However, the role of immunotherapy in the treatment of non-metastatic PCa is controversial,^28^ and further studies are needed on this subject.

There are also studies in the literature investigating CD4+ T lymphocyte counts and their prognostic importance in various malignancies. Tredan et al evaluated 204 patients with breast cancer and suggested that the decrease in the CD4+ T lymphocyte count might be associated with poor overall survival.^29^ In another study evaluating metastatic breast cancer, Yang et al^30^ reported that CD4+ T lymphocytes constituted a negative independent risk factor and had both predictive and prognostic value for progression-free survival.^30^

The main limitation of the current study is that changes in lymphocyte subtypes were not evaluated in the routine follow-up after treatment. In addition, as described in the literature, lymphocyte infiltration values in prostatectomy specimens are likely to contribute to our clinical evaluation. However, this should be the subject of another study. Another limitation of our study is the short follow-up period. This may have resulted in not clearly demonstrating the role of lymphocyte subtypes in clinical progression and biochemical recurrence. Despite the stated limitations, we consider that the decrease in the CD4+ T type of lymphocytes in peripheral blood with the increasing PSA level and pathological stage may become a prognostic marker of PCa and play a role in the treatment decision to be taken afterward. In this respect, we believe that our study will guide future research.

In conclusion, we determined that the percentage of CD4+ T lymphocytes in the peripheral blood samples of patients with PCa decreased with the increasing pathological stage and PSA level before surgical treatment. In this respect, we believe that the evaluation of the CD4 T lymphocyte count in the peripheral blood has a prognostic importance and may contribute to the management of the disease.

## Figures and Tables

**Table 1.** Demographic and Clinicopathological Characteristics of the Patients

Age (Years)	Min-Max (median)	48-77 (64)
	*Mean ± SD*	64.14 ± 6.25
Body mass index (kg/m^2^)	Min-Max (median)	20.28-37.04 (27.43)
	*Mean ± SD*	27.69 ± 3.47
		**n (%)**
PSA (ng/mL)	*Min-Max (median)*	1.4-158 (8.2)
	*Mean ± SD*	15.61 ± 25.23
cT stage	**1**	65 (47.4)
	**2**	72 (52.6)
D’Amico risk classification	**Low risk**	36 (26.3)
	**Intermediate risk**	60 (43.8)
	**High risk**	41 (29.9)
TRUS biopsy GS		
GS 3+3		48 (35)
GS 3+4		43 (31.4)
GS 4+3		15 (10.9)
GS 4+4		16 (11.7)
GS 4+5		11 (8.1)
GS 5+5		4 (2.9)
TRUS rate	*Min-Max (median)*	0.1-100 (20)
	*Mean ± SD*	28.61 ± 28.18
RRP pathology GS		
GS 3+3		23 (16.8)
GS 3+4		44 (32.1)
GS 3+5		1 (0.8)
GS 4+3		24 (17.5)
GS 4+4		21 (15.3)
GS 4+5		15 (10.9)
GS 5+4		7 (5.1)
GS 5+5		2 (1.5)
ISUP 2014		
Grade 1		23 (16.8)
Grade 2		44 (32.1)
Grade 3		24 (17.5)
Grade 4		22 (16.1)
Grade 5		24 (17.5)
pN status	**Nx**	76 (55.5)
	**N0**	57 (41.6)
	**N1**	4 (2.9)
RRP pathology tumor area	Min-Max (median)	0.5-100 (15)
	Mean ± SD	22.31 ± 22.24
Surgical margin	**Negative**	**n (%) **108 (78.8)
	**Positive**	29 (21.2)
Pathological stage	**T2**	64 (46.7)
	**T3**	73 (53.3)
	**T3a**	49 (35.8)
	**T3b**	24 (17.5)
Biochemical recurrence	**Absent**	101 (73.7)
	**Present**	36 (26.3)
Time to recurrence (months)	Min-Max (median)	1-24 (9)
	Mean ± SD	9.90 ± 6.73
Clinical progression	**Absent**	134 (97.8)
	**Present**	3 (2.2)
Time to progression (months)	Min-Max (median)	3-10 (4)
	Mean ± SD	5.67 ± 3.79
Adjuvant therapy	**Absent**	102 (74.5)
	**Present**	35 (25.5)
Follow-up duration (months)	Min-Max (median)	2-24 (13)
	Mean ± SD	12.81 ± 6.20

cT, clinical T; GS, Gleason score; PSA, prostate-specific antigen; RRP, retropubic radical prostatectomy; SD, standard deviation; TRUS, transrectal ultrasound.

**Table 2.** Distribution of Lymphocyte Subtypes in the Peripheral Blood of the Patients

CD 3+ (T lymphocyte) %	Min-Max (median)	42.2-89.6 (74.1)
	Mean ± SD	72.65 ± 8.92
CD 19+ (B lymphocyte) %	Min-Max (median)	1.1-23.3 (9.4)
	Mean ± SD	10.23 ± 4.47
CD 16/56+ (NK)%	Min-Max (median)	1.4-55.6 (14.2)
	Mean ± SD	16.12 ± 9.13
CD 3+/CD 4+ (helper T lymphocyte) %	Min-Max (median)	19.7-73.3 (42.9)
	Mean ± SD	43.46 ± 9.90
CD 3+/CD 8+ (cytotoxic T lymphocyte) %	Min-Max (median)	5.8-69.7 (25.9)
	Mean ± SD	27.52 ± 9.91
Total lymphocyte %	Min-Max (median)	9.9-75.7 (27.8)
	Mean ± SD	28.42 ± 9.91

**Table 3.** Correlation Between Lymphocyte Subtypes and Age, BMI, PSA, and Gleason Score

	CD3+	CD19+	CD16/56+	CD3+/CD4+	CD3+/CD8	Total Lymphocyte Ratio
	*r*/*p*	*r*/*p*	*r*/*p*	*r*/*p*	*r*/*p*	*r*/*p*
Age	−0.152^c^	−0.145^c^	0.233^d^	−0.172^c^	0.111^c^	−0.067^c^
	0.077	0.091	**0.006**	**0.044**	0.195	0.434
BMI	−0.117^c^	0.059^c^	0.084^d^	0.019^c^	−0.128^c^	0.053^c^
	0.174	0.491	0.331	0.830	0.135	0.538
Gleason score	−0.003	−0.135	0.080	0.107	−0.163	0.063
	0.974	0.117	0.354	0.215	0.057	0.465
PSA	−0.096	−0.026	0.075	−0.196	0.070	0.005
	0.263	0.759	0.384	**0.022**	0.419	0.950

BMI, body mass index; PSA, prostate-specific antigen.

^c^ *r* = Pearson’s correlation coefficient.

^d^ *r* = Spearman’s correlation coefficient.

^*^ *P* < .05; ^**^ *P* < .01.

**Table 4.** Relationship of Lymphocyte Subtypes with Clinical and Pathological Features

	CD3+	CD19+	CD16+/56+	CD3+/CD4+	CD3+/CD8+	Total Lymphocyte Ratio
	*P*	*P*	*P*	*P*	*P*	*P*
Pathological stage T2 (n = 64) T3 (n = 73)	.114^a^	.280^a^	.079^b^	**.048** **^a,^** *	.702^a^	.667^a^
Biochemical recurrence Present (n = 36) Absent (n = 101)	.529^a^	.288^a^	.667^b^	.692^a^	.755^a^	.648^a^
Surgical margin Positive (n = 29) Negative (n = 108)	.487^ a^	.818	.246^ b^	.909^ a^	.628^ a^	.663^ a^
Adjuvant therapy Present (n = 35) Absent (n = 102)	.878^ a^	.709^ a^	.584^ b^	.824^ a^	.591^ a^	.202^ a^
Cancer upgrade Present (n = 60) Absent (n = 77)	.228^a^	.075^ a^	.627b	.591a	.229a	.565a

^a^Student’s *t*-test; ^b^Mann–Whitney *U* test.
